# SARS-CoV-2 immunogenicity in individuals infected before and after COVID-19 vaccination: Israel, January–March 2021

**DOI:** 10.1017/S0950268821001928

**Published:** 2021-08-17

**Authors:** Kamal Abu Jabal, Hila Ben-Amram, Karine Beiruti, Ira Brimat, Ashraf Abu Saada, Younes Bathish, Christian Sussan, Salman Zarka, Michael Edelstein

**Affiliations:** 1 Ziv Medical Centre, Safed, Israel; 2 Bar-Ilan University, Safed, Israel

**Keywords:** COVID-19, Israel, SARS-CoV-2, vaccine immunogenicity, vaccines

## Abstract

Between December 2020 and March 2021, we measured anti-SARS-CoV-2 IgG titres among 725 Israeli hospital workers vaccinated against COVID-19. Infection post-dose 1 vaccination did not increase IgG titres, and individuals infected post-dose 1 had IgG levels comparable to never-infected individuals who received a single dose, lower than fully vaccinated, never-infected individuals. This suggests dose 2, currently not offered to those infected post-dose 1, may be required in these individuals. Larger studies should confirm whether individuals infected post-dose 1 need the second.

## Background

As of May 2021, Israel was the country with the highest COVID-19 vaccine coverage [1]. As of 2 June 2021, approximately 56% of the population had received two doses of the BNT162b2 mRNA vaccine [2]. The impact of vaccination on SARS-CoV-2 transmission is now clearly seen, with a basic reproduction ratio (R_0_), remaining below 1 since March 2021 [2] despite a gradual easing of social distancing measures since then.

In Israel, individuals previously infected with SARS-CoV-2 were not eligible for vaccination until early March 2021. Evidence shows that memory B cells persist for at least 8 months [3]. Vaccinating previously infected individuals with one dose of BNT162b2 vaccine generates a boost-type IgG response up to 10 months post-infection [4]. Countries including Israel now recommend a single dose of vaccine in infected individuals [5]. The benefit of the second dose in such individuals remains uncertain.

Vaccine efficacy from one dose of BNT162b2 vaccine in uninfected individuals begins 14 days post-vaccination and reaches 50–80% between days 15–35 [6]. Therefore, a proportion of patients will be infected within the first 14 days post-vaccination. In Israel, these individuals are not offered a second dose, based on the assumption that one dose confers immunity in infected individuals. However, immunogenicity in patients infected after one vaccine dose has not been evaluated.

We serially measured anti-spike IgG response in a cohort of vaccinated healthcare workers according to SARS-CoV2 infection status (never infected or infected before or after vaccination) in order to inform vaccination policy.

## Methods

All employees of Ziv Medical Centre (ZMC), a hospital in Northern Israel, were offered the BNT162b2 vaccine from December 2020. Before vaccination, nucleocapsid (N) IgG antibody levels were measured in consenting workers, using a highly sensitive and specific SARS-CoV-2 IgG qualitative assay (Abbott, Abbot Park, USA) [7], followed by a quantitative LIAISON SARS-CoV-2 S1/S2 IgG assay (DiaSorin, Saluggia, Italy) for verification purposes [7]. Workers with detectable IgG antibodies at baseline and/or evidence of a previous positive PCR test SARS-CoV-2 were considered previously infected.

AntiSARS-CoV-2 spike IgG levels were measured around 21 days (range 15–35 days) post-dose 1 and around 51 days (range 41–65 days) post-dose 1 (corresponding to 30 days post-dose 2 for those who received two doses), using the LIAISON Diasorin SARS-CoV-2 S1/S2 IgG assay [7].

All workers who developed COVID-19-compatible symptoms during the study period (December 2020–March 2021) were PCR tested. Individuals with a positive PCR test were classified as infected post-vaccination.

Antibody levels were reported using geometric mean concentration (GMC) alongside 95% confidence intervals (95% CI). In the Israeli context, gender, ethnicity and time elapsed between infection and vaccination were not significantly associated with IgG levels [4] and were therefore not adjusted for. Anti-SARS-CoV2 spike IgG antibody levels were compared among groups of ZMC workers according to the number of doses received (1 or 2) and their infection status (uninfected, infected prior to vaccination, infected after vaccination), using Kruskal–Wallis tests. The study was approved by ZMC's ethics committee (0133–20-ZIV).

## Results

Of approximately 1500 employees, 725 received at least one dose of vaccine and were tested at least once post-vaccination. Of these, 25 had evidence of pre-vaccination infection and 35 had evidence of infection post-dose 1 (Table 1), of which 32 before they were eligible for dose 2. These 32 individuals did not receive dose 2 as they were no longer eligible owing to their infected status. Not all these patients were tested twice (Table 1). Of the 541 individuals tested 15–35 days post-dose 1, 96.9% had detectable antibodies and 99.8% of those tested 41–65 days post-dose 1 had detectable antibodies.

**Table 1.** Geometric mean concentrations of anti-SARS-CoV-2 spike IgG antibodies among healthcare workers who received the BNT162b2 mRNA COVID-19 vaccine, Israel, December 2020 to March 2021

		1st serological test (15–35 days post-vaccine dose 1)					2nd serological test (41–65 days post-vaccine dose 1)				
Groups	*n*	*n*	Median age	Median days from dose 1	GMC (AU/ml)	95% CI	*n*	Median age	Median days from dose 1	GMC (AU/ml)	95% CI
Previously infected 1 dose	6	16^a^	42	21	773.4	449.1–1331.8	3	42	47	464.7	17.7–12 168.3
Previously infected 2 doses	19						16	42	50	570.6	380.6–855.6
Infected after dose 1	32	9	41	21	73.3	56.3–95.4	20	46	50	83.8	60.5–115.9
Infected after dose 2	3	–		–	–	–	2	45	56	230.2	22.9–2308.9
Uninfected 1 dose	22	516^b^	45	21	64.1	60.3–68.2	3	51	54	90.2	4.7–1721.7
Uninfected 2 doses	643						492	46	52	196	189–203.2
Overall	725	541	45	21	69.3	64.6–74.5	536	45	52	196.1	187.7–205

a Includes individuals who received one or two doses in total.

b Includes individuals who received one or two doses in total.

**Fig. 1.**
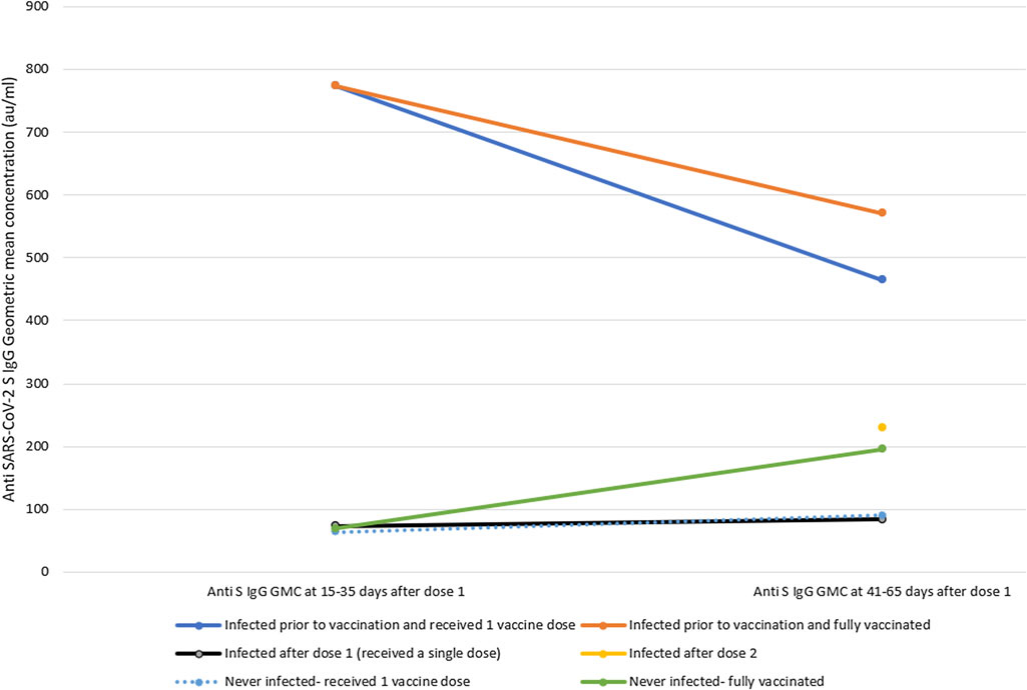
Anti SARS CoV-2 IgG levels among Israeli Healthcare workers according to infection status and number of doses received, January-March 2021.

Compared with uninfected individuals, previously infected individuals had high IgG titres at 21 days post-vaccine that remained high approximately 50 days post-vaccination (Table 1 and Fig. 1). Those previously infected and fully vaccinated had IgG titres more than twice higher than previously uninfected individuals who received two doses (GMC 570.6 *vs.* 196, *P* < 0.001, Table 1). Only three previously infected individuals who received a single dose were tested for IgG at 50 days post-vaccination. Their GMC was high but not statistically significantly different from those uninfected who received two doses (GMC 464.7 *vs.* 196, *P* = 0.2, Table 1).

Among those previously infected who received a second dose, IgG titres 50 days post-dose 1 (approximately 30 days post-dose 2) had not increased compared with pre-dose 2 (GMC 570.6 *vs.* 773.4, *P* = 0.27, Table 1 and Fig. 1) and were not significantly higher than those who received a single dose (570.6 *vs.* 464.7, *P* = 0.8, Table 1).

Among the 32 patients who tested PCR-positive post-dose 1 and before being eligible for dose 2, nine were serologically tested around 21 days post-vaccination, of which eight were infected within 14 days of being vaccinated and one between days 14 and 21. Among the nine, IgG levels at 21 days were comparable to those never infected (GMC 73.3 *vs.* 64.3, *P* = 0.21, Table 1 and Fig. 1) and more than 10 times lower than those infected prior to vaccination (GMC 73.3 *vs.* 773.4, *P* < 0.001, Table 1). At 50 days post-vaccination, IgG titres among the 20 patients infected post-dose 1 tested at this time point were 83.8 (95% CI 60.5–115.9), comparable to those previously uninfected who received a single dose (GMC 90.2, *P* = 0.27), and lower than either those uninfected who received two doses (GMC 196, *P* < 0.001), or those infected prior to vaccination (GMC 552.4, *P* < 0.001). Among the small number (*n* = 3) of patients infected post-dose 2, IgG titres were comparable to those never infected (GMC 230.2 *vs.* 196, *P* = 0.38, Table 1).

## Discussion

In this small cohort, participants infected with SARS-CoV-2 pre-vaccination had a strong and persisting response after one BNT162b2 dose. Dose 2 in those individuals had no impact on IgG titres. Several countries, including France and Israel, recommend one vaccine dose for individuals previously infected with COVID-19. Although the number of individuals in our cohort who received one dose only and were serologically tested at 50 days is very small, our data support this policy. Baseline serology was qualitative only and it was therefore not possible to describe the influence of baseline IgG titres on future immunogenicity or viral load on those subsequently infected.

Our study shows that when it comes to vaccinating infected individuals, the sequence of events matters. Individuals in our cohort infected post-vaccination had IgG titres at 21 and 50 days similar to those never infected who received the same number of doses and much lower than those infected pre-vaccination. These data suggest that individuals infected after a single dose of the BNT162b2vaccine should remain eligible for a second dose to ensure adequate protection levels. Although the sample size of this study was too small to warrant a policy change by itself, larger studies should be conducted to confirm or refute these results in order to change vaccine policy if needed.

Our study presents limitations. First, our numbers are small. Notably, most previously infected who received one dose were not tested again at 50 days. Very few patients were infected after receiving their second dose, a finding compatible with the high effectiveness of the vaccine [8]. Second, post-vaccination testing was symptom-based, and we could not detect asymptomatic patients infected post-vaccination. Third, our study focuses on the BNT162b2 vaccine and it is possible that these findings are not relevant to other vaccines. Our study was conducted when the wild type and the alpha variant were co-circulating in Israel. The findings may not be applicable to variants that subsequently emerged, notably the delta variant, against which the effectiveness of the BNT162b2 vaccine is reduced [9]. Lastly, our study measures circulating antibody levels and does not measure the functionality of these antibodies through a neutralisation assay, nor other components of the immune system such as T-cell activity.

## Conclusion

This study demonstrates no benefit in terms of IgG titres in receiving a second dose among individuals infected prior to vaccination, and more importantly, that infection after vaccination (dose 1 or 2) seems to have very little effect on immunogenicity as measured by circulating IgG levels. It is therefore important to not assume that individuals infected after their first dose are fully immune. Larger immunogenicity studies, as well as efficacy studies, should confirm or refute the need for a second dose of COVID-19 vaccine in these individuals, in particular in the context of emerging variants against which vaccines are less effective.

## Data Availability

The anonymised dataset used for this study is available for academic purposes by request to the corresponding author.
